# Contralateral approach to a carotid bifurcation aneurysm in a case of multiple intracranial aneurysms: a case report

**DOI:** 10.1186/1757-1626-2-35

**Published:** 2009-01-09

**Authors:** Seyed Ali F Tabatabai, Ali Tayebi Meybodi, Mohammad Hashemi, Zohreh Habibi

**Affiliations:** 1 Department of Neurosurgery, Imam Khomeini Hospital, Tehran University of Medical Sciences, Tehran, Iran

## Abstract

**Background:**

Traditionally, surgery of the anterior circulation aneurysms of the cerebral vasculature is dictated by the site of the lesion, excluding such midline lesions as anterior communication artery aneurysms. Few reports address the issue of using a single craniotomy to obliterate multiple aneurysms located in both hemispheres.

**Case presentation:**

A 51 year-old Caucasian right handed housewife lady (weight 61 kg, height 159 cm) presented with a headache of acute onset which proved to be caused by acute subarachnoid hemorrhage. Cerebral computed tomographic angiography revealed multiple aneurysms. The patient underwent a right pterional craniotomy to obliterate right middle cerebral, distal basilar and left carotid bifurcation aneurysms. The post-operative course was uneventful.

**Conclusion:**

Despite technical difficulties of approaching cerebral vasculature through a contralateral craniotomy, this policy is advised in selected cases in which the benefits of unilateral craniotomy outweigh the risks of brain retraction.

## Background

Although controversy exists on the surgical treatment of multiple intracranial aneurysms, some authors advocate surgery of all multiple aneurysms due to the bleeding tendency of these lesions overtime. Successful management of such a malady via a single craniotomy, would be fruitful regarding the morbidity and mortality [1,2].

Previous studies have addressed the contralateral approach to the anterior circulation aneurysms, yet clarification of the very guidelines regarding microsurgical techniques and neuroanesthetic aspects is still evolving [2-4]. The present case is an example of contralateral pterional craniotomy used to clip an ICA bifurcation saccular aneurysm.

## Case presentation

A 51-year-old Caucasian left-handed housewife lady (weight 61 kg, height 159 cm) was admitted to our institution because of severe sudden onset headache followed by transient loss of consciousness and vomiting. On examination she was fully conscious and oriented and complained of severe headache. Nuchal rigidity was evident. No focal neurological deficit was found. Temperature was 38°C and other vital signs were stable. Past medical history was negative and she did not consume any medication.

**Figure 1:**
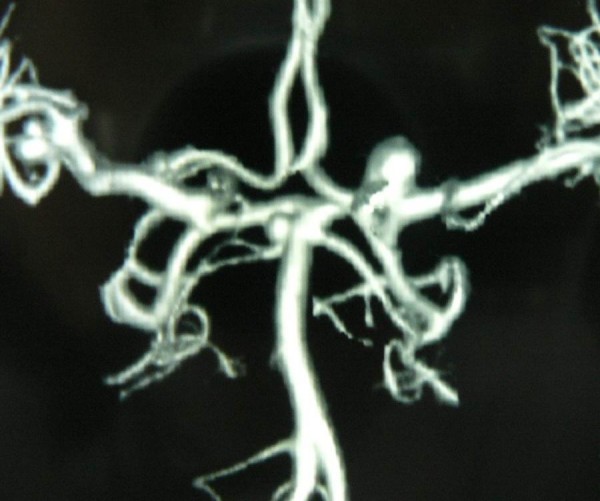
**Three-dimensional reconstructed CT angiography of brain vasculature revealing multiple aneurysms: Right MCA, Left ICA bifurcation, and distal basilar artery**.

CT scan of the head revealed acute subarachnoid hemorrhage prominently occupying the right sylvian fissure. CT angiography was performed (figure 1). The cerebral vasculature was found to harbor three saccular aneurysms at: (1) right MCA trifurcation, (2) left ICA bifurcation, and (3) distal basilar artery. The right MCA aneurysm was presumed to be the ruptured aneurysm due to location of subarachnoid clot and irregular shape of the aneurysm.

**Figure 2:**
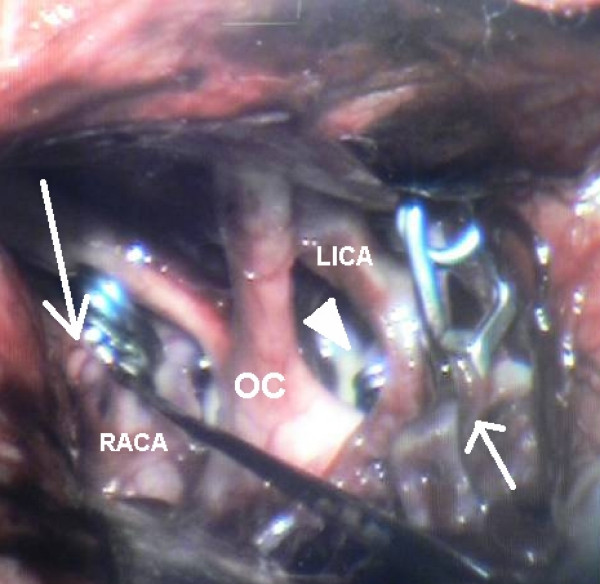
**Intraoperative photograph of right pterional exposure showing clipping right MCA (small arrow), left carotid bifurcation (large arrow) and distal basilar (arrowhead) aneurysms**. LICA: Left Internal Carotid Artery, OC: Optic Chiasm, RACA: Right Anterior Cerebral Artery.

A right pterional craniotomy was performed with the patient in the supine position and head rotated 30 degrees to left. After elevation of the craniotomy flap, the sphenoid ridge was drilled flush with the orbital roof to facilitate exposure of the basilar tip aneurysm. After duratomy, gentle frontal lobe retraction allowed CSF drainage from the optic and carotid cisterns. Right optic nerve and right ICA were identified and the Sylvian fissure was opened from medial to lateral, following the bifurcation of right ICA to MCA trifurcation. A saccular aneurysm was found in the MCA trifurcation projecting inferolaterally. After dissection of the neck it was clipped. Next, opening the membrane of Liliequist, let to the distal basilar aneurysm from the corridor between the right optic nerve and right ICA. The aneurysm was located between the right PCA and right SCA with a wide neck, and its dome projected laterally to right. Successful clipping was achieved. The left ICA bifurcation aneurysm was identified after following the right A1, anterior communicating and left A1 arteries with minimal brain retraction, and gaining benefit from favorable neuroanesthesia. The aneurysm projected superiorly and was also clipped (figure 2).

**Figure 3:**
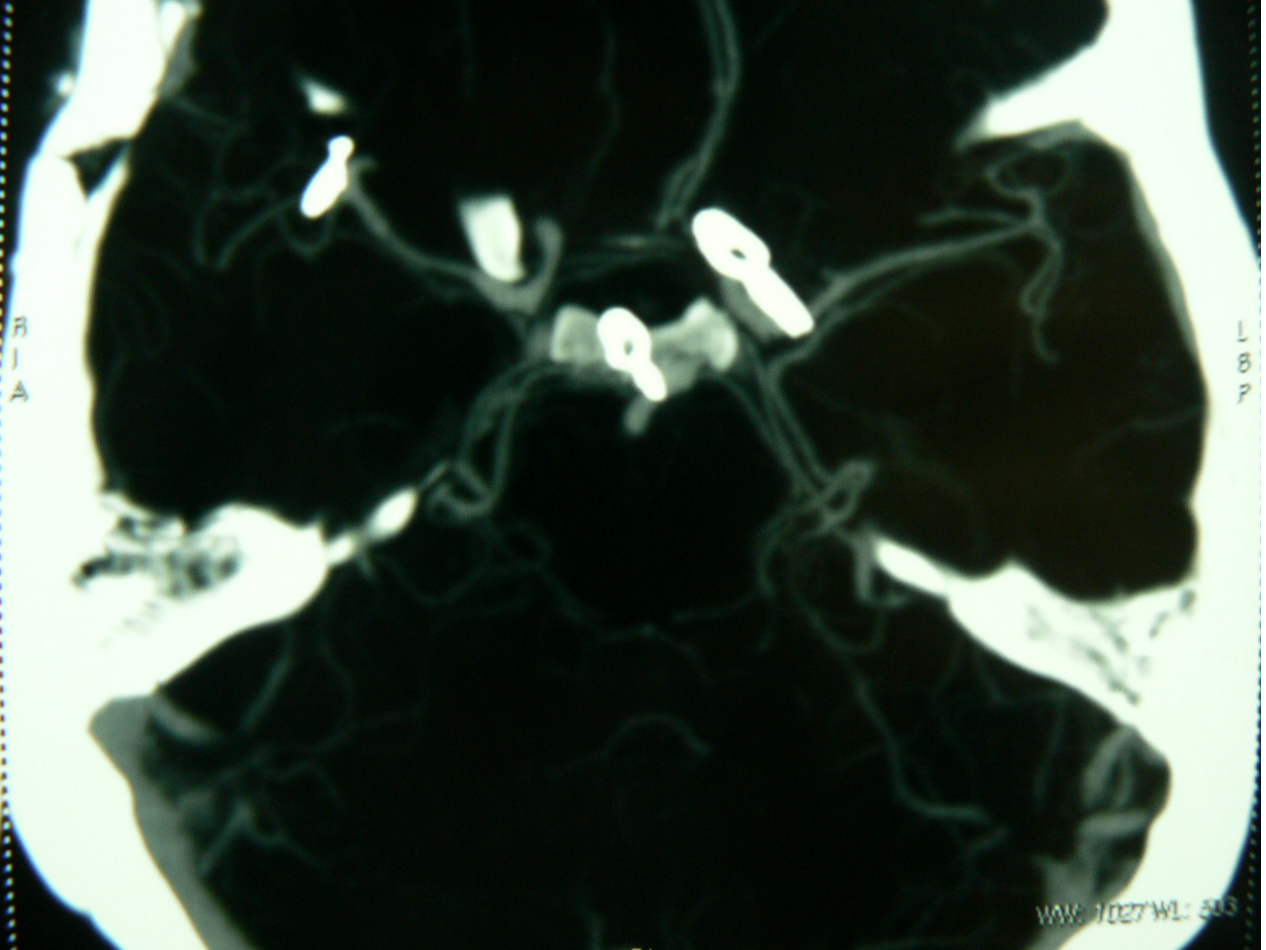
**Postoperative CT angiogram of brain showing successful obliteration of all aneurysms**.

The patient experienced an uneventful post-operative period and was discharge within 5 days of surgery. A follow-up CT angiography confirmed successful obliteration of all lesions and preservation of normal cerebral vasculature (figure 3).

## Conclusion

Clipping of multiple intracranial aneurysms via a single craniotomy could be a safe and reasonable strategy, provided the microsurgical anatomical aspects of individual patients are addressed. Patient selection, dexterity of the surgeon, and fulfillment of neuroanesthetic measures are important factors to be considered in this issue.

## Abbreviations

CT: Computed Tomography; CSF: Cerebrospinal Fluid; ICA: Internal Carotid Artery; MCA: Middle Cerebral Artery

## Consent

Written informed consent was obtained from the patient for publication of this case report and accompanying images. A copy of the written consent is available for review by the Editor-in-Chief of this journal.

## Competing interests

The authors declare that they have no competing interests.

## Authors' contributions

SAFT is the attending surgeon and revised the manuscript. ATM prepared the manuscript and MH and ZH reviewed and revised the final manuscript.
